# Burn image segmentation based on Mask Regions with Convolutional Neural Network deep learning framework: more accurate and more convenient

**DOI:** 10.1186/s41038-018-0137-9

**Published:** 2019-02-28

**Authors:** Chong Jiao, Kehua Su, Weiguo Xie, Ziqing Ye

**Affiliations:** 1 School of Computer Science, Wuhan University, Wuhan, 430072 China; 2 Institute of Burns, Wuhan Hospital No. 3 and Tongren Hospital of Wuhan University, Wuhan, 430060 China

**Keywords:** Burn image, Deep learning, Mask R-CNN, Image segmentation

## Abstract

**Background:**

Burns are life-threatening with high morbidity and mortality. Reliable diagnosis supported by accurate burn area and depth assessment is critical to the success of the treatment decision and, in some cases, can save the patient’s life. Current techniques such as straight-ruler method, aseptic film trimming method, and digital camera photography method are not repeatable and comparable, which lead to a great difference in the judgment of burn wounds and impede the establishment of the same evaluation criteria. Hence, in order to semi-automate the burn diagnosis process, reduce the impact of human error, and improve the accuracy of burn diagnosis, we include the deep learning technology into the diagnosis of burns.

**Method:**

This article proposes a novel method employing a state-of-the-art deep learning technique to segment the burn wounds in the images. We designed this deep learning segmentation framework based on the Mask Regions with Convolutional Neural Network (Mask R-CNN). For training our framework, we labeled 1150 pictures with the format of the Common Objects in Context (COCO) data set and trained our model on 1000 pictures. In the evaluation, we compared the different backbone networks in our framework. These backbone networks are Residual Network-101 with Atrous Convolution in Feature Pyramid Network (R101FA), Residual Network-101 with Atrous Convolution (R101A), and InceptionV2-Residual Network with Atrous Convolution (IV2RA). Finally, we used the Dice coefficient (DC) value to assess the model accuracy.

**Result:**

The R101FA backbone network gains the highest accuracy 84.51% in 150 pictures. Moreover, we chose different burn depth pictures to evaluate these three backbone networks. The R101FA backbone network gains the best segmentation effect in superficial, superficial thickness, and deep partial thickness. The R101A backbone network gains the best segmentation effect in full-thickness burn.

**Conclusion:**

This deep learning framework shows excellent segmentation in burn wound and extremely robust in different burn wound depths. Moreover, this framework just needs a suitable burn wound image when analyzing the burn wound. It is more convenient and more suitable when using in clinics compared with the traditional methods. And it also contributes more to the calculation of total body surface area (TBSA) burned.

## Background

Burn injuries require immediate treatment by estimating the burn area and burn depth. Normally, this work is hard to solve by the general nurse or the doctor.

### Current calculation methods

**Fig. 1:**
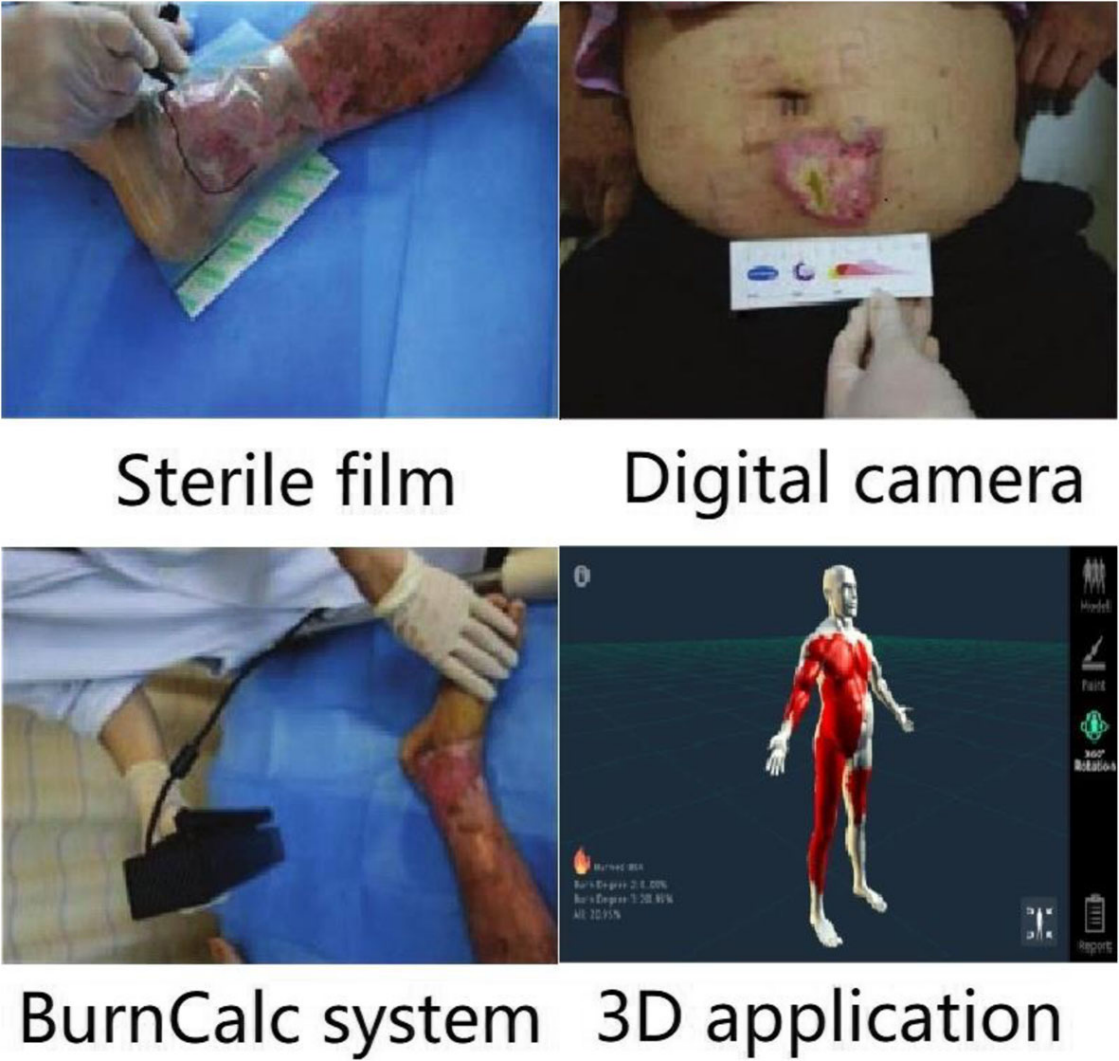
Current mainstream diagnostic methods of burn wounds

As shown in Fig. 1, the current assessment of a burn wound consists of three main methods [1–4]. The first method is to attach the sterile film to the wound of the patient, then draw the wound boundary with a marking pen, and finally calculate the area of the wound. This method is influenced by the subjective factors of the marking person and will bring about errors in some depths. The second method is to use the digital camera to calculate the wound area based on the principle of camera imaging. Then, this digital camera uses NIH ImageJ software to calculate the wound area. However, this software cannot automatically identify the edge of the wound, which makes it necessary to label the wound edge before calculating the wound area. The third method is to use the BurnCalc system [4]. The doctor uses this system to build 3D models of patients through specialized 3D scanning equipment, then draws the wound on the 3D model using a special drawing software, and finally calculates the wound area. This method needs to draw the artificial wound, which causes the final area calculation result to be highly inaccurate. In 2018, Cheah et al. designed a 3D application to calculate the burn area [5]. This application builds the 3D body model by the people’s height and weight. Then, the doctors mark the burn area on the surface of the 3D model. After marking, the application can calculate the burn area automatically. However, this calculation relies on the precise burn area marked. Moreover, accurately marking the burn area is a time-consuming work which is not suitable for busy first-aid work.

We can conclude from above that the complicated and time-consuming part in these methods is to determine the wound region from the patient’s skin, which is where many errors occur. To solve this problem, we used state-of-the-art deep learning techniques to segment the wound region, and this method can be well applied to the calculation of the wound area.

### Object detection

Analyzing object recognition and location in images is one of the most fundamental and challenging problems in computer vision. In the deep learning, Regions with Convolutional Neural Network (R-CNN) [6] is an object detection method in imagery analysis. This system consists of three parts. The first part generates category-independent region proposals by selective search [7]. The second part is a large convolutional neural network that extracts a fixed-length feature vector from each region. The last part is a set of class-specific linear support vector machines (SVM).

In order to realize faster training and evaluating, the Fast R-CNN [8] obtains feature vectors from the shared feature map. To avoid region proposals distortion, it adds a region of interest (RoI) pooling layer [8] on the basis of the R-CNN. Moreover, the author used the multi-task loss in the classification and regression to accelerate the system. In 2016, the Faster R-CNN [9] applied the region proposal network (RPN) [9] network to accelerate the speed of the generation of category-independent region proposals. With the powerful deep learning baseline, the object detection results can reach a high standard.

### RPN

In the early object detection system, the generation of region proposals is exhaustive search. Then, in 2013, Uijlings et al. proposed a selective search to relieve the computational pressure [7]. Ren et al. then proposed an RPN which generates high-quality region proposals from the shared feature maps [9]. In generation, the RPN used a small sliding window on the shared feature map. Then, at each sliding-window location, the RPN generated multiple scale rectangle boxes on the original image, and these rectangle boxes are called anchors [9]. After that, the RPN network used a small convolutional neural network to predict the object score and box regression at each anchor. Then, the RPN sorted the score of each anchor and used the greedy non-maximum suppression to obtain region proposals.

### Residual network (ResNet)

ResNet [10] was proposed in 2016 by Kaiming et al. In the convolutional neural network, the deeper the network is, the more features it can obtain. Although this feature of the convolutional neural network leads to a series of breakthroughs in image classification, it also brings a lot of new problems. For example, as the network depth deepens, the notorious problems of vanishing/exploding gradient occur. The deep residual learning network, which adds a reference on each layer to learn the residual function, can address the degradation problem properly. The main networks in the ResNet are the ResNet101 [10] and ResNet50 [10].

### Feature pyramid network (FPN)

FPN [11] was proposed in 2017 by the Lin et al. This network can build high-level semantic feature maps at all scales. It contains three parts which are bottom-up, top-down, and lateral connections [11]. In general, ResNet is the backbone network of the FPN. The output feature map of each convolution layer is denoted as C_2_, C_3_, C_4_, and C_5_ in the bottom-up part. The top-down part then upsamples the feature map by using a factor of 2, and the upsampled map is merged with the corresponding bottom-up map. Then a 3 × 3 convolution is appended on each merged map to generate the final feature map. The final outputs of feature maps are called P_2_, P_3_, P_4_, and P_5_. In this article, we adopt the ResNet101 and Atrous [12] in FPN, and we called this backbone network as R101FA.

### Mask R-CNN

In principle, Mask R-CNN is an intuitive extension of Faster R-CNN, yet building the mask branch properly is important for good results. Mask R-CNN is different from the Faster R-CNN in three points. The first point is that the Mask R-CNN adds a mask branch to predict the category of every pixel in the region proposals. The second point is that the use of the RoIAlign [13] to achieve the pixel-pixel alignment between network inputs and outputs. The third point is the definition of the mask loss. For each RoI associated with ground-truth class k, mask loss is only defined on the *k*-th mask (other mask outputs do not contribute to the loss).

## Method

In this article, we used a novel method for employing the state-of-the-art deep learning framework Mask R-CNN. For a more refined result and a faster training speed, we modified the Mask R-CNN to adapt to our dataset. We changed the loss function of the class branch and adopted the Atrous Convolution [12] in the backbone network. In order to make a better segmentation result, we tried several mainstream backbone networks, and the R101FA demonstrated the best segmentation results.

### Data set

From December 2017 to June 2018, we worked with the burn department of the Wuhan Hospital No. 3. Ethics approvals were granted by the Wuhan Hospital No. 3 and Tongren Hospital of Wuhan University. The patients used in this research have already signed the informed consent.

**Fig. 2:**
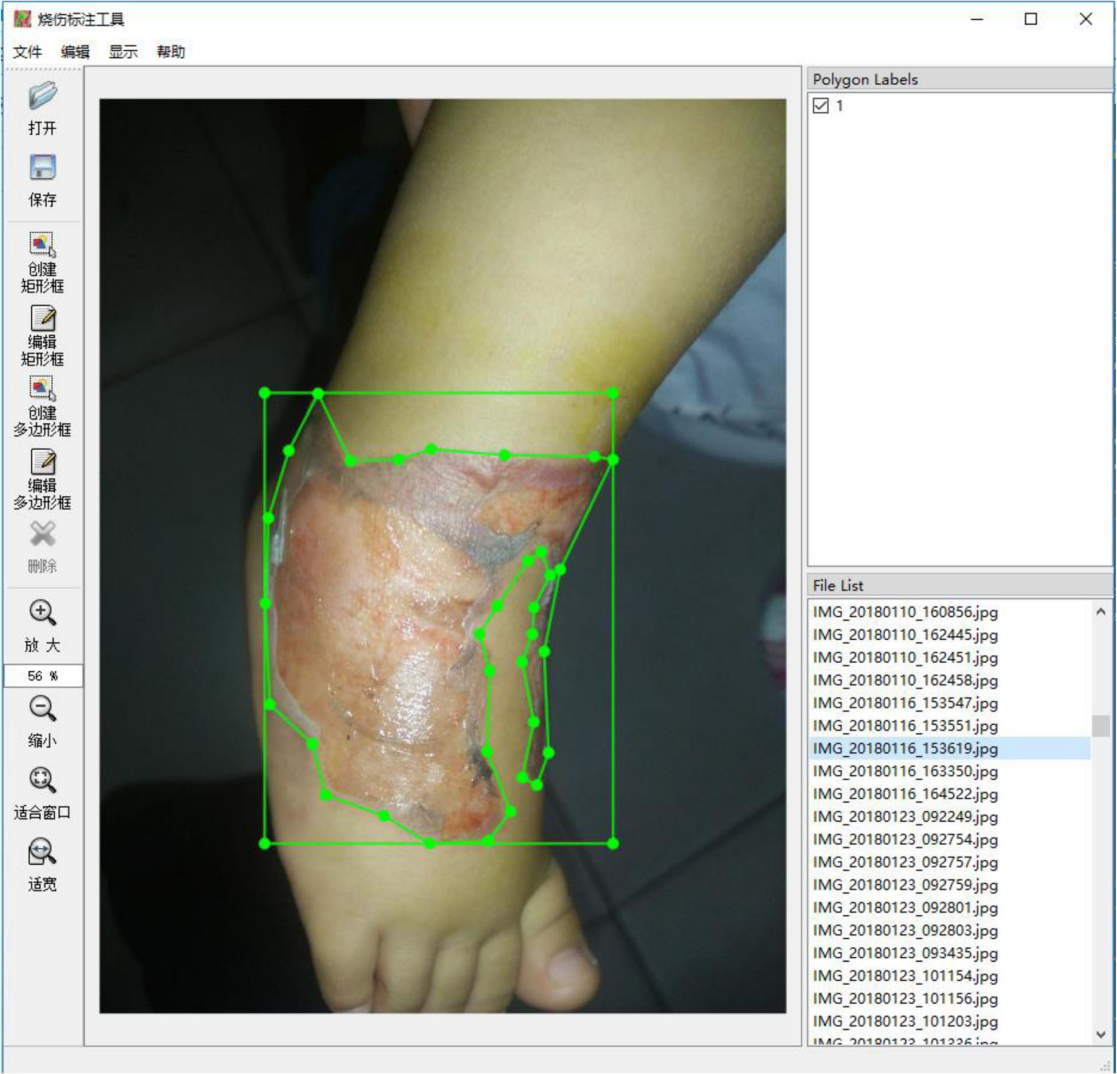
Annotation tool. With the help of professional doctors, we use the corresponding annotation tool to annotate the image data. This annotation tool is made with QT (a C++ framework)

In order to obtain enough data, we used our smartphone to collect images of fresh burn wounds in the hospital every day. Then, we used our own software to annotate burn images and saved the marked content in Common Objects in Context (COCO) data set format. Figure 2 shows the software which we used to annotate. In order to ensure the accuracy of this framework, we annotated the burn images carefully under the guidance of the professional doctors and avoided mistaking confusing parts such as gauze and blood stains as wounds. With the help of doctors and nurses, we finally annotated 1000 burn images for training and another 150 for evaluating.

### Network architecture

**Fig. 3:**
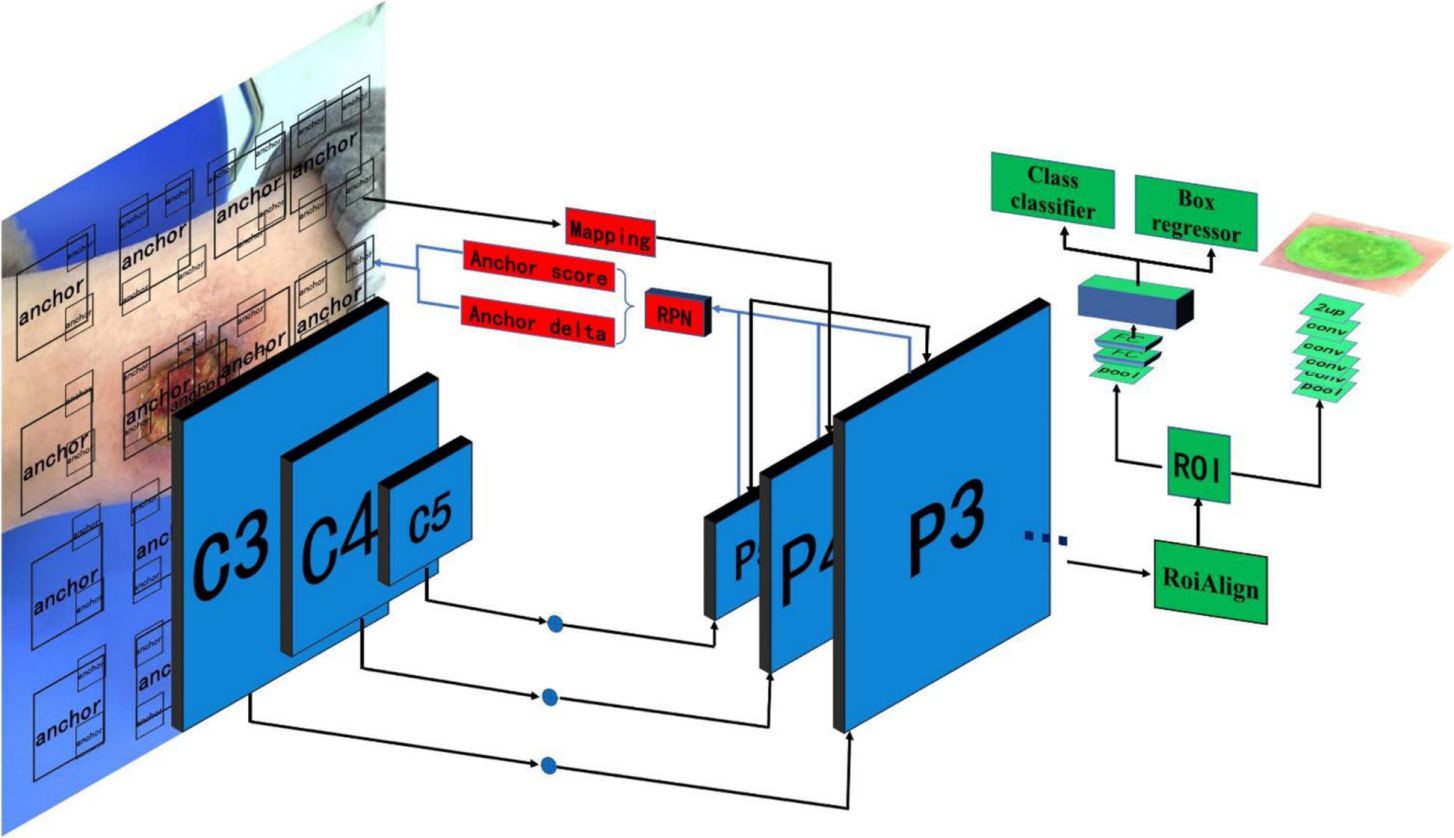
Block diagram of the burn image segmentation framework. The framework contains three parts. The blue part is extracting the features from the image. The red part is the region proposal network to generate the regions of interests. The green part is the network heads to classify and regress

As shown in Fig. 3, our framework contains three parts. The first part is the backbone network to extract the feature maps. The second part is the RPN [9] network to generate the RoI [9]. Finally, we process the object detection and mask prediction from each RoI. Because there is only one category (here, we do not consider the depth of burn wound), we changed the loss function of the mask branch and classification branch to fit our data set. In the process of training, we collected almost all kinds of burn wound images to train our model, totaling 1000 after filtering. At the same time, in order to realize faster training speed and less evaluating time, we tried different backbone networks in our framework. Finally, we used the R101FA as the backbone network of our framework.

**Fig. 4:**
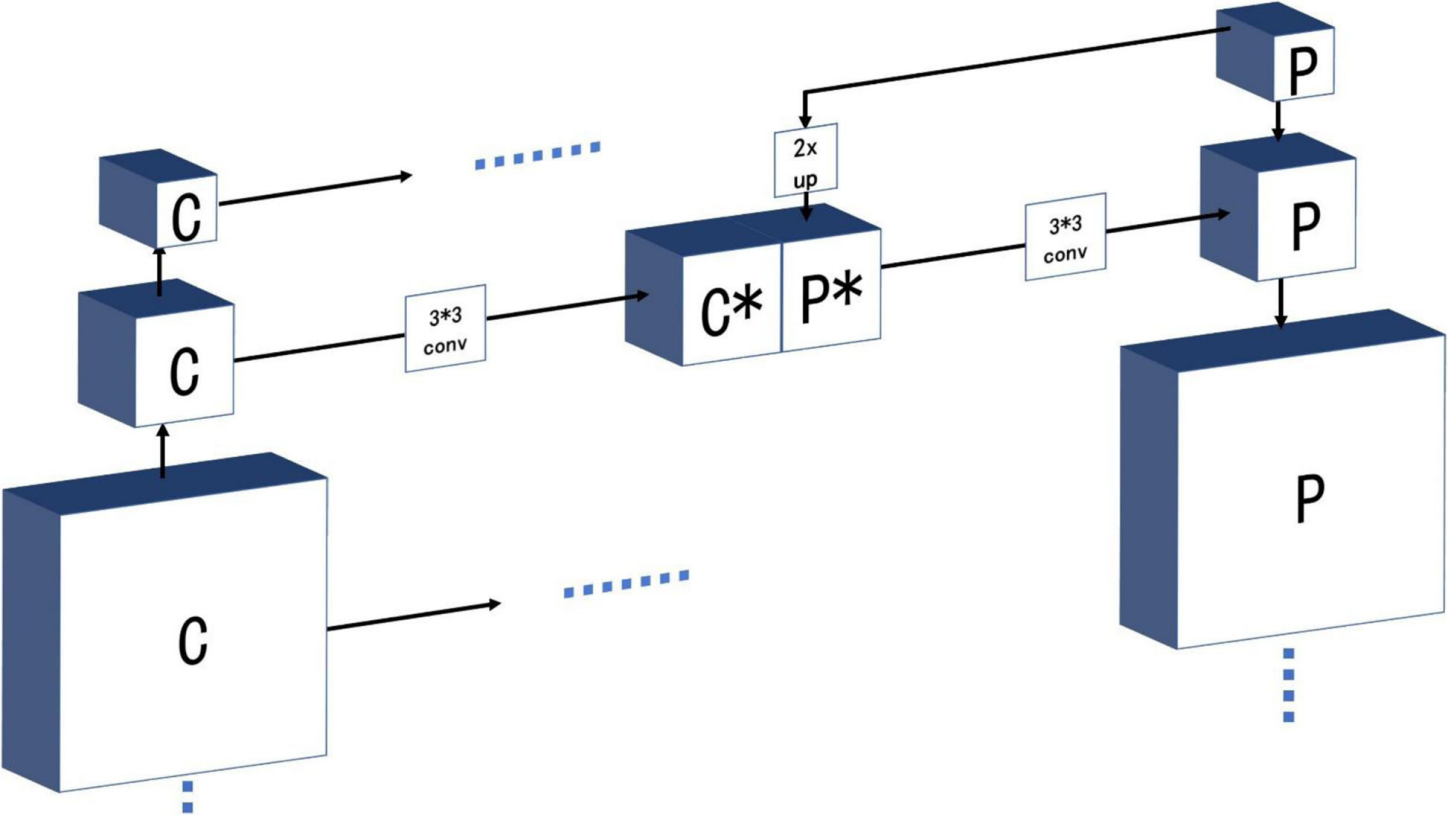
The process of generating feature map. The left column is the bottom-up of the feature pyramid network (FPN). The right column is the top-down of the FPN. The middle column is the lateral connection of the FPN

In this article, our backbone network is based on the R101FA. The ResNet101 is made up of 101 layers. We use C_1_, C_2_, C_3_, C_4_, and C_5_ to define these output feature maps. As shown in Fig. 4, we obtain final feature maps P_2_, P_3_, P_4_, and P_5_.

Here, we use a 1 × 1 convolution kernel to get the first feature map P_5_ by undergoing the output of the C_5_. Then, we upsample the P_5_ to get the P^*^ and produce C^*^ by the 3 × 3 convolution kernel undergoing the C_5_, and the P_4_ map is the result of merging C^*^ with P^*^. After iterating all C, we can build P_2_, P_3_, P_4_, and P_5_.

### Atrous

**Fig. 5:**
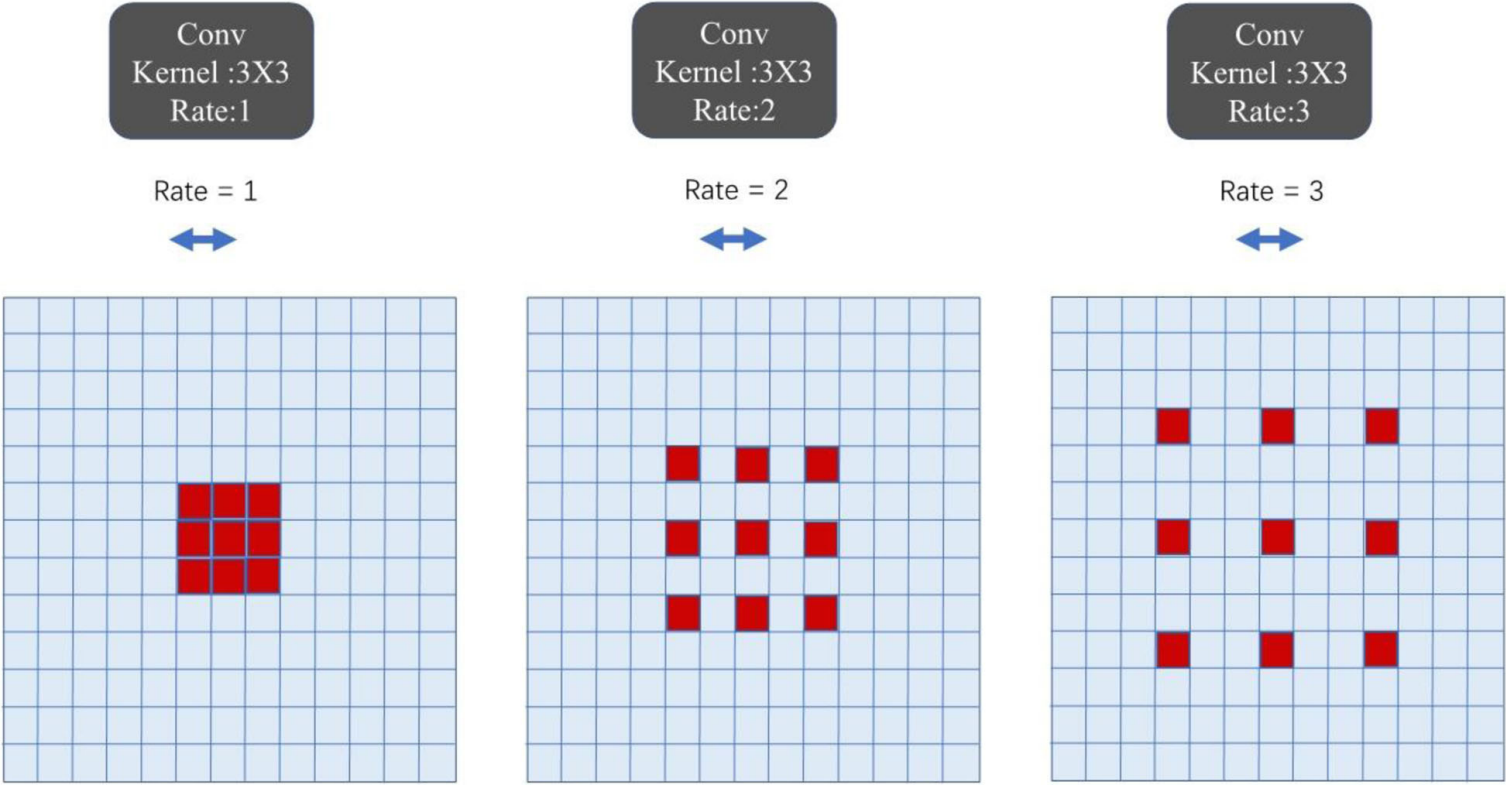
Atrous convolution. Atrous convolution with kernel size 3 × 3 and different rates. From left to right in the figure, the atrous rates are 1, 2, and 3. Standard convolution corresponds to atrous convolution with rate = 1. In this article, we set the rate as 2

In the convolutional neural network, we employ atrous convolution [12] in ResNet. The traditional convolution kernel is usually composed of a dense matrix of N × N. The kernel of atrous convolution is no longer a dense matrix, and it is shown by Fig. 5 that different rates represent different convolution kernels. Compared with traditional convolution kernels, employing a larger value of atrous rate enlarges the model’s field-of-view, enabling object encoding at multiple scales. This structure is suited for our burn dataset which consists of varying burn depths and burn sizes. In this research, we set the rate at 2.

### RPN in FPN

We adopt RPN in FPN to propose the candidate of region proposals. The detail is different from the original RPN network. The original RPN network just adopts one feature map, but in our network, we build several feature maps. In order to handle the images easier, we resized the images to 1024 × 1024 and filled the image with zero to prevent distortion. In order to contain all possible rectangular boxes, we defined five scales 32 × 32, 64 × 64, 128 × 128, 256 × 256, and 512 × 512. Every scale has three aspect ratios 0.5, 1, and 2. It was not necessary to define all scales on every feature map; we just defined one scale per feature map. Here, in order to correspond five scales, we added P_6_ on the basis of P_5_ and it is the output of the max-pooling after the P_5_. Hence, according to this idea, we can generate all possible rectangular boxes (anchor [9]) on the original image.

**Fig. 6:**
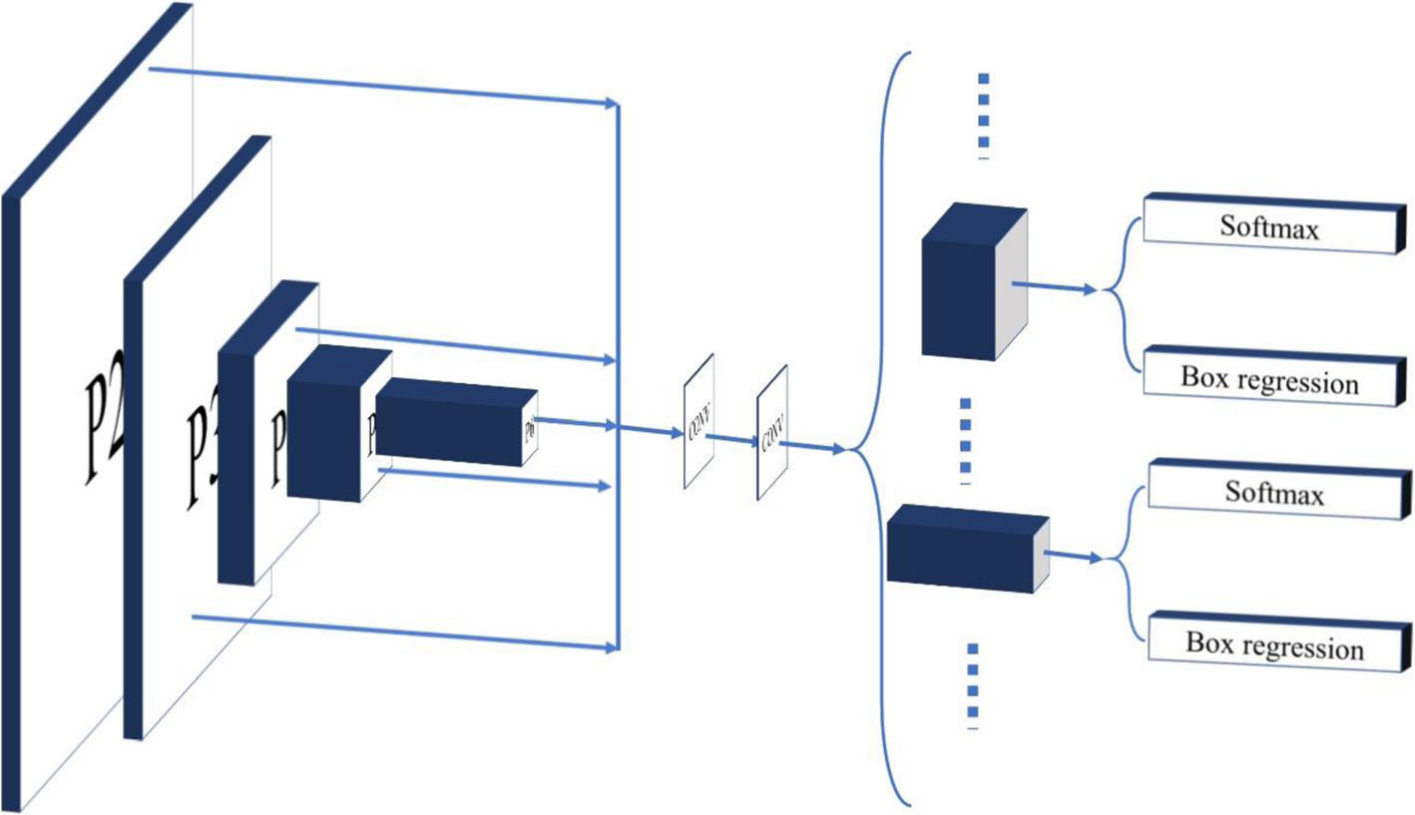
The detail of the region proposal network. The left column is the output of the feature extracting. The middle content is the convolutional neural network. The right column is to classify and regress

In the RPN network, we filter N numbers of RoIs by a small convolution network. This small network determines the object possibility of each anchor, and we call this possibility anchor score. We sorted all the anchors by this score and take the top N high score boxes as RoI. Moreover, in order to adjust the position of each anchor, the small network also predicts the regression offsets of each anchor. Therefore, in FPN, there are several feature maps and this small network is shared with all feature maps, and the detail is shown in Fig. 6.

### RPN training

As shown in Fig. 6, the outputs of the RPN network are score and regression offsets of each anchor. Here, we define two loss functions to train the RPN network. The first is the score loss *L*_rpnScore_and the second is regression loss *L*_rpnReg_.

To calculate *L*_rpnScore_, we assign two kinds of labels which are the positive label and the negative label to each anchor. The anchor which has an intersection over union (IOU) overlap higher than 0.7 with any ground-truth bounding box is a positive label, and the anchor which has an IOU overlap lower than 0.3 with all ground-truth boxes is a negative label. Here, in order to ensure that all the ground-truth boxes correspond to at least one anchor, we will label the highest IOU anchor with each ground-truth box as a positive label. Therefore, we can get all the positive and negative anchors. We encode these anchors into a sequence of 0 and 1, and the sequence is the objective output in the RPN target judgment. As it is shown in Fig. 6, we apply the *softmax* function to the output of the RPN to get target possibility for all anchors. And then, we use the cross-entropy function to calculate the *L*_rpnScore_.

Then, we apply a linear function to the output of the RPN network and predict the regression parameters (*t*^∗^). We calculate the regression offsets (*t*) of each positive anchor. The regression offsets are the same as [8], and it contains four values (*x*, *y*, *w*, *h*). *x* and *y* are the offset ratios of the positive anchors’ center point based on the associated ground-truth boxes center point. Then, *w* and *h* are the logarithmic values of the aspect ratio of positive anchors and the associated ground-truth boxes. Finally, we used the *smooth*_L1_ to calculate the *L*_*rpnReg*_ which is shown in Eq. 1. Here, we stipulate that just the positive anchor will contribute the *L*_*rpnReg*_.

1 $$ {L}_{rpnReg}\left({t}_i\right)=\frac{1}{N_{r\mathrm{eg}}}{\sum}_i{p}_i^{\ast }{L}_{r\mathrm{eg}}\left({t}_i,{t}_i^{\ast}\right) $$

Here, *i* is the index of an anchor in the mini-batch and ${p}_i^{\ast }$ is 1 if the anchor is positive; otherwise, p_i_^*^ is 0. Here, *t*_*i*_ and ${t}_i^{\ast }$ are the four vectors representing the regression offset, and *t*_*i*_ represents regression offset of a positive anchor based on the associated ground-truth box. And ${t}_i^{\ast }$ represents the predicted regression offset. The regression loss function is shown in Eq. 2. The *smooth*_L1_ is defined in Eq. 3.

2 $$ {L}_{r\mathrm{eg}}\left(t,{t}^{\ast}\right)={\sum}_{i\in x,\mathrm{y},\mathrm{w},\mathrm{h}}s{\mathrm{mooth}}_{L_1}\left(t-{t}_i^{\ast}\right) $$

3 $$ s{\mathrm{mooth}}_{L_1}(x)=\left\{\begin{array}{c}0.5\ {x}^2\kern2.55em \mathrm{if}\ \left|x\right|<1\\ {}\left|x\right|-0.5\kern2.25em \mathrm{otherwise}\end{array}\right. $$

We used Eq. 4 to make a detailed explanation for regression offset.

4 $$ {\displaystyle \begin{array}{cc}\begin{array}{c}{t}_x=\frac{x-{x}_a}{w_a}\\ {}{t}_w=\log \left(\frac{w}{w_a}\right)\end{array}& \begin{array}{c}{t}_y=\frac{y-{y}_a}{h_a}\\ {}{t}_h=\log \left(\frac{h}{h_a}\right)\end{array}\\ {}\begin{array}{c}{t}_x^{\ast }=\frac{x^{\ast }-{x}_a}{w_a}\\ {}{t}_w^{\ast }=\log \left(\frac{w^{\ast }}{w_a}\right)\end{array}& \begin{array}{c}{t}_y^{\ast }=\frac{y^{\ast }-{y}_a}{h_a}\\ {}{t}_h^{\ast }=\log \left(\frac{h^{\ast }}{h_a}\right)\end{array}\end{array}} $$

After choosing the RoIs from the anchors, we map the RoIs on the feature map for subsequent operation of the framework. But in our framework, we have four feature maps. Unlike generating anchors, we do not make each RoI correspond to a feature map. Considering that P_2_ contains all image features, we map all RoIs to P_2_. After mapping, the three parallel branches handle the mapping results.

### Loss function

In our framework, our loss contains five aspects. The RPN network contains two losses. In the parallel branches, there are three losses. We define the three losses as *L*_mCls_, *L*_mBReg_, and *L*_mMask_. Therefore, our final loss is L =*L*_rpnScore_+*L*_rpnReg_+*L*_mCls_+*L*_mBReg_+ *L*_mMask_.

#### Class loss

In Mask R-CNN, the author applied *softmax* on the output of the fully connected layer and used the cross-entropy function to calculate the class loss. This method was applied to solve the multi-class classification tasks. But in our task, our goal is simply to segment the burn wounds. Therefore, we used two classifiers to replace the multiple classifiers. We applied the *sigmoid* function on the output and used the cross-entropy function to calculate the loss. We used *y* to define the ground-truth of the N RoIs. The output of *sigmoid* is y^*^. Then, the *L*_mCls_ is Eq. 5.

5 $$ {L}_{\mathrm{mCls}}=\frac{1}{N}{\sum}_i^N\left({y}_i\ast \left(-\mathit{\log}{y}_i^{\ast}\right)+\left(1-{y}_i\right)\ast \left(-\log \left(1-{y}_i^{\ast}\right)\right)\right) $$

#### Bounding-box loss

As mentioned above, the RPN network will predict the regression offset of each anchor. In the box branch, the input will be the coordinate of RoI. These coordinates are the result of the RoI that applied the regression offset of the RPN network. We then used the same way as *L*_*rpnReg*_ to calculate the *L*_mBReg._

#### Mask loss

In Mask R-CNN, the author applied a small FCN [14] network on the RoI. And in the mask branch, the author predicted the m × m mask. The mask is the output of the sigmoid function which is applied to each pixel. Then, the author calculated the mask loss according to the mask class to avoid the competition between classes and used the binary cross-entropy to define the loss.

However, in this article, we just calculated the mask loss of the positive RoI and did not use the idea of competition between classes. Moreover, we defined the size of each ground-truth mask and predicted the mask as 28 × 28 to reduce memory consumption. Hence, the ground-truth RoI was scaled to 28 × 28 and padded with zero to avoid distortion. In the output of the mask branch, we will scale each RoI to the same size to calculate the mask loss.

#### Regularization loss

As mentioned above, we collected little data sets. And in order to prevent over-fitting of the model, we add the loss of regular term for entire loss function. We can see the details from the formula 6

6 $$ {L}_{regLoss}=\lambda {\sum}_{i=1}^n\left({W}_i^2\cdotp \frac{1}{N_{w_i}}\right) $$

This is the L2 regularization loss which represents the weight decay and aims to reduce the weight values to fit data well. In the formula, *W*_*i*_ is the weight values of the *i*-th layer and ${N}_{w_i}$ is the size of the *W*_*i*_. The *λ* is a hyper-parameter which is set as 0.0001 here.

### Training detail

In order to obtain better training results, we did not randomly initialize weight in this framework. The initialization of the weight includes two parts. In the convolutional neural network, we used the pre-trained COCO model to initialize our backbone network. In the network head, we used the Gaussian distribution to initialize the weight values. Similar to the transfer learning, we fine-tune the convolutional neural network of our framework by collecting data.

Moreover, we tried several convolutional networks to extract the feature map from the original image. These backbone networks are Residual Network-101 with Atrous Convolution (R101A), Residual Network-101 with Atrous Convolution in Feature Pyramid Network (R101FA), and InceptionV2-Residual Network with Atrous Convolution (IV2RA). Through the experiment, we find the R101FA backbone has the best segmentation result. Before training, the images are resized to a 1024 width for a proper input in the network. Then, similar to the [10], the input data will undergo five convolution layers C_1_, C_2_, C_3_, C_4_, and C_5_ which have strides of 2, 4, 8, 16, and 32 related to the input image.

After extracting a feature map, the RPN network handled the output of the backbone network. First, the RPN network generated N anchors (the anchor scale is for the original image) on the center of the sliding window. Then, we calculated the IOU value (per anchor) to judge if the anchor is positive or negative. As in [8, 9], each image has N-sampled RoIs which have a ratio of 1:3 of positive to negatives. Then, we pooled each positive anchor to a fixed size. After that, we connected a fully connected network to extract a 2048 dimensions feature vector. This vector is used for the classifier and the box regressor. At the same time, the RoIs will undergo two convolution layers, then we predicted the image mask.

### Burn area calculation

Burn area calculation is an important part of burn diagnosis. The framework as mentioned above is an auxiliary technique for calculating burn area and has great significance for fast, convenient, and accurate burn area calculation. As it is shown in Fig. 1, for example, the second method needs to manually mark the edge of the burn wound when calculating. Similar to the 3D application, it is not conducive for rapid treatment of patients. However, if we combine our segmentation framework with this software, we can get a more efficient and convenient area calculation tool. In a sense, we can apply our framework to the calculation of burn wound area.

In our plan, we intend to combine the 3D modeling and mesh parameterization technology with our segmented framework which calculates the burn wound area. The calculation mainly consists of three steps:

- Step-1: Building a 3D model of patient pictures through 3D reconstruction technology.
- Step-2: Mapping the 3D model to the planar domain by mesh parameterization algorithm.
- Step-3: Segmenting the burn regions by using our framework and calculate the total body surface area (TBSA) value.

Some 3D reconstruction technologies are already very mature, such as BodyTalk reconstruction system [15] and Kinect reconstruction system [16], which makes the 3D model building process easier. The mesh parameterization algorithm such as RiccFlow [17] and Authalic [18] make the second step easier to implement. Hence, our segmentation framework can achieve a faster, easier, and more accurate area calculation.

## Results

### Burn characterization

**Fig. 7:**
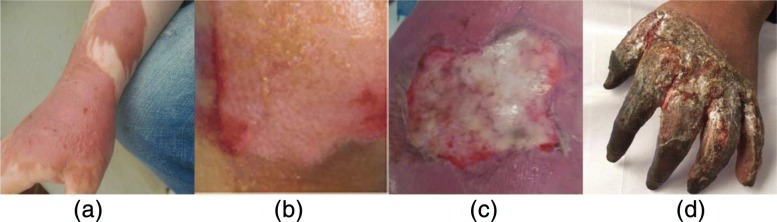
**a**–**d** Different burn depth. From left to right is superficial, superficial thickness, deep partial thickness, and full-thickness burn

There are four main depths of burn wounds: (i) superficial dermal burn, (ii) superficial partial thickness burn, (iii) deep partial thickness burn [19], and (iv) full-thickness burn. Figure 7 shows the four depths of burns across four images.

In the past, image processing techniques for total or partial segmentation often use evolving curvilinear boundaries because of their adaptive capacity and modeling of the internal structures of the images [19, 20]. In this article, we come to the conclusion that the burn does not exhibit uniform boundaries. Moreover, various depths of wounds make the segmentation work harder. The traditional technologies no longer work in the burn segmentation if we want to segment all burn situations. Hence, we adopt the deep learning framework to realize the segmentation.

### Segmentation results

In this paper, our framework mainly segments the burn wounds without classifying the depth of the wound. However, in order to show the stability and generalization ability of our work, we selected 150 images to evaluate our method. With the help of professional doctors, we combined these images from the different burn area size images and different burn depth images.

### Segment different sizes

**Fig. 8:**
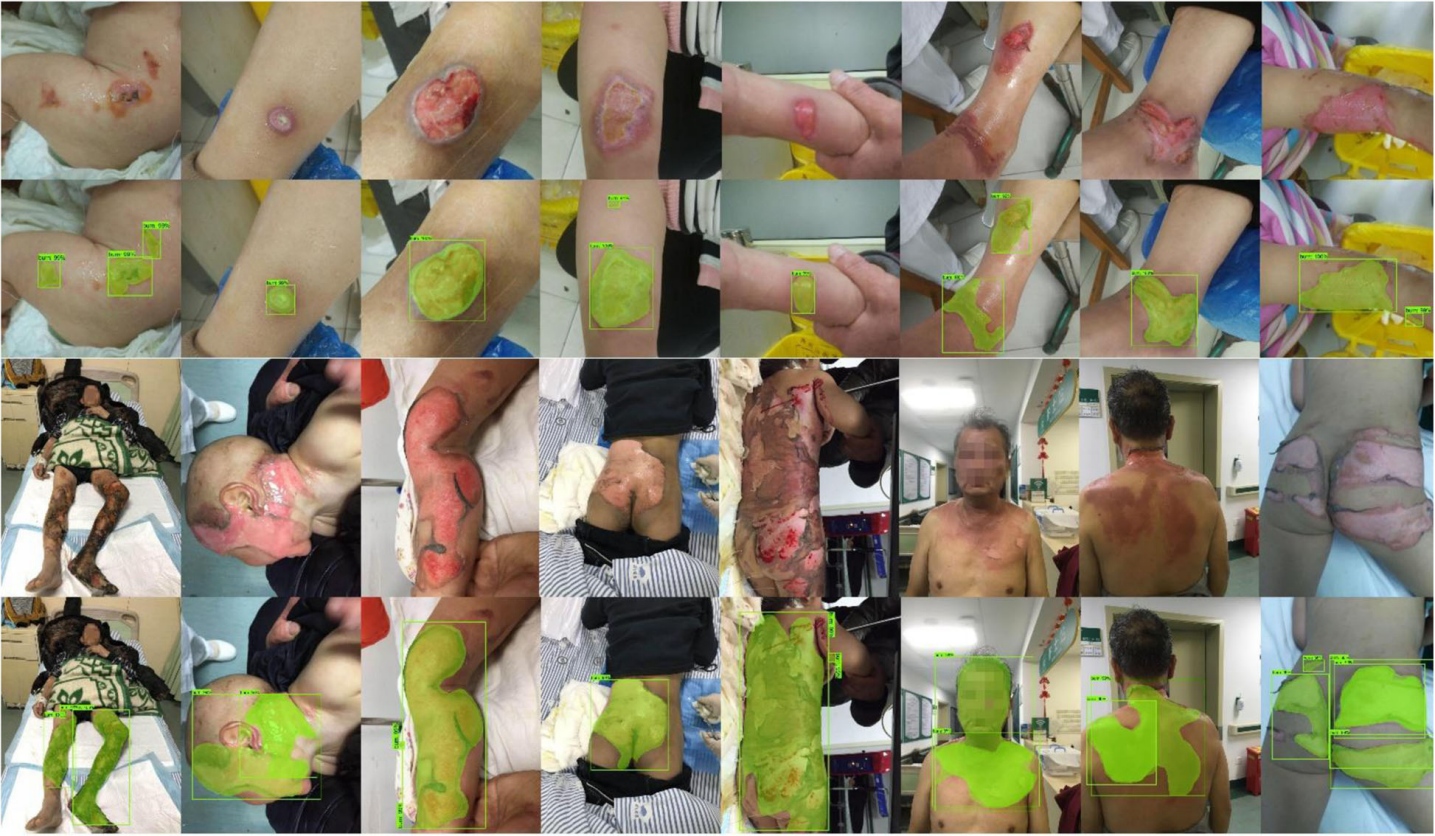
Segmentation results of different burn wounds sizes. The first and second rows show the %TBSA*<* 5% wounds and the segmentation results of R101FA. The third and fourth rows show the %TBSA*>* 20% wounds and the segmentation results of R101FA (*R101FA* residual network-101 with atrous convolution in feature pyramid network*. TBSA* total body surface area)

The first advantage is that our model can segment the different sizes of the burn wounds. Through the experiment, our model expressed high robustness of the different burn wound sizes. We chose the four depths of burn wounds. As it is shown in Fig. 8, our model performed fine segmentation in the %TBSA < 5% burn wound. Moreover, for the large burn area, our model also performed very well. This is also shown in Fig. 8.

### Segment different depth

**Fig. 9:**
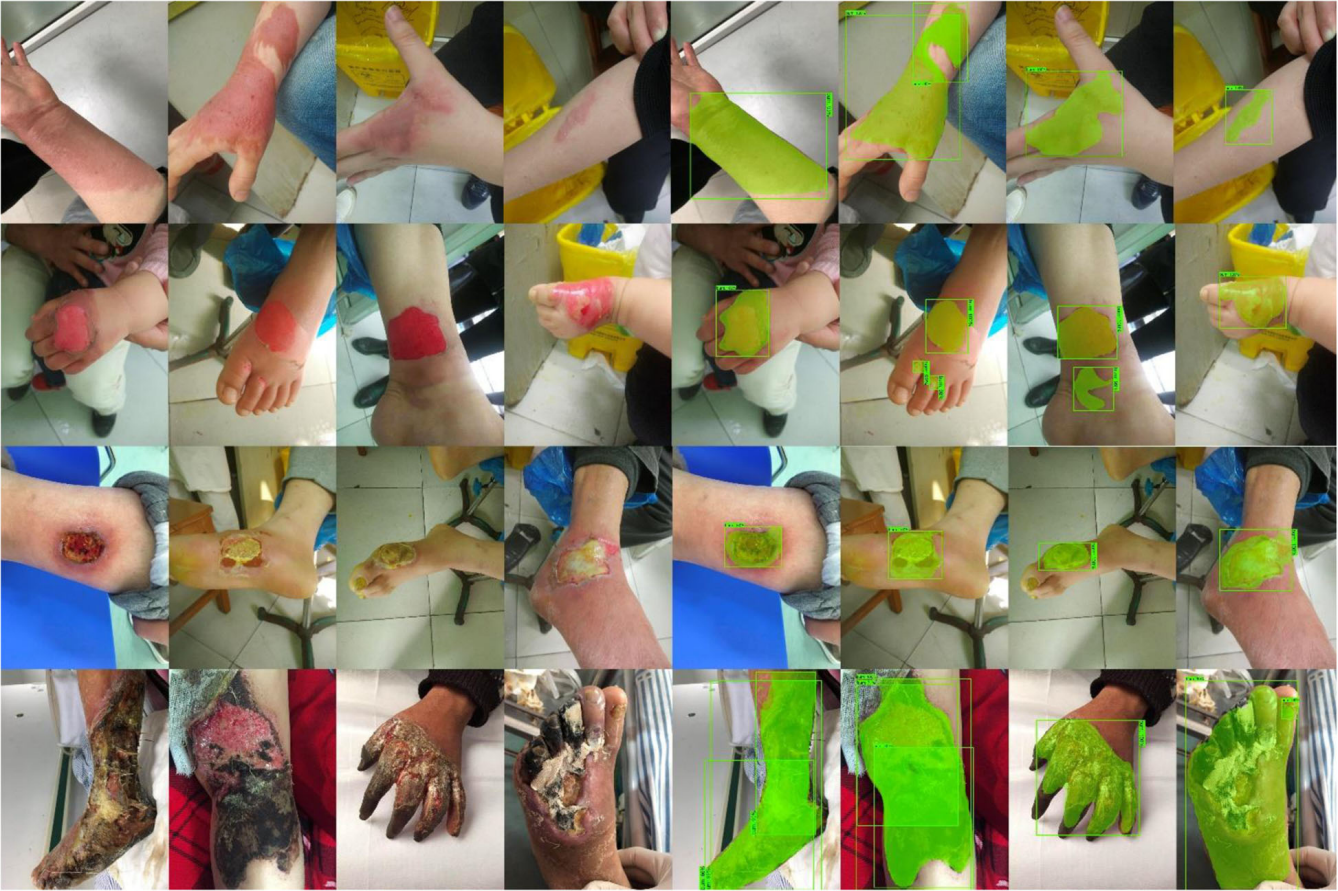
Segmentation results of different burn wound depths. These four lines are the superficial, superficial thickness, deep partial thickness, and full-thickness burn from top to bottom

There are many reasons to cause a burn such as hydrothermal fluid, high-temperature gas, and flame. In addition, these reasons can lead to different burn wounds depths. Because of the variance of each depth of burn, it increases the difficulty of segmentation. However, in our model, we can segment the different burn wound depths successfully. Figure 9 shows the segmentation results of different depth burns.

### Method comparison

We compared our method with traditional methods and modern methods.

#### Traditional methods

**Fig. 10:**
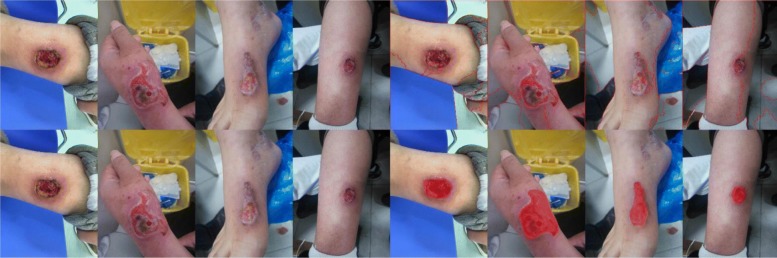
Traditional methods compared. The first line is the result of the watershed algorithm and the second line is the result of R101FA (*R101FA* residual network-101 with atrous convolution in feature pyramid network)

In the image segmentation, the traditional methods always use the edge features or spectral feature of the image to accomplish the segmentation. In this article, we tried the Watershed Algorithm [21] for our burn images. The Watershed Algorithm is based on the edge of the image and also labels the spectral feature on the image. Most of the time, this algorithm uses the color histogram to decide which color is to be the watershed. But in the burn images, there are many colors similar to the burn wound. This element made it difficult for the watershed algorithm to segment the burn wounds. We finally tried a different parameter to get better segmentation. As shown in Fig. 10, we can see that the Watershed Algorithm could not show good segmentation in a complex picture environment.

#### Modern methods

In recent years, the image segmentation method which is based on deep learning achieves excellent performance. In this article, therefore, we chose different architectures to be the backbone network in our framework. These backbone networks are IV2RA [22], R101A [10], and R101FA.

**Fig. 11:**
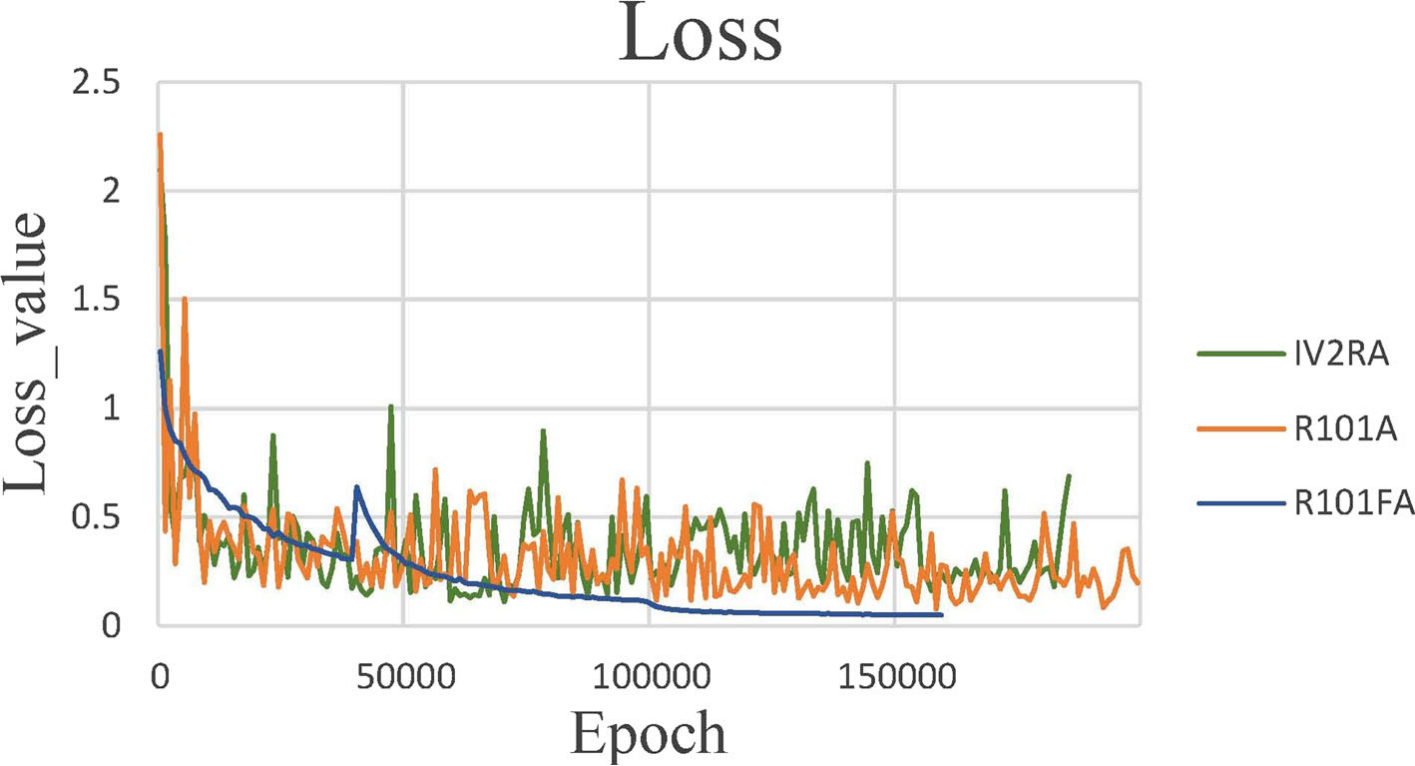
Loss results of different backbone networks. *IV2RA* inceptionV2-residual network with atrous convolution, *R101A* residual network-101 with atrous convolution, and *R101FA* residual network-101 with atrous convolution in feature pyramid network

In training, for the best training effect, we set 20 epochs for IV2RA and R101A. But in our method, we set 16 epochs. Each epoch contained 1000 iterations. First, we will show the loss reduction of the different backbone networks. As shown in Fig. 11, we see that our method can achieve a better loss reduction than two of the other backbone networks. Moreover, our method used fewer epochs than the other backbone networks.

During the evaluation, we chose 150 burn images to evaluate the different backbone networks. Here, because we only considered the wound segmentation effect, there was no need to use the mean average precision to evaluate the model. Hence, we chose the Dice coefficient (DC) [23] to evaluate the percentage of segmentation accuracy from the ground truth. The DC measures the concordance between two enclosed areas. The formula is as follows:

7 $$ \mathrm{DC}\%=100\frac{2 TP}{FP+2 TP+ FN} $$

In detail, the number of false positives is the FP value. The false positive represents the incorrectly segmented pixels. The FN is the number of false negatives. The false negative represents the target pixels that are not segmented. TP is the true positive. The true positive represents the correct segmentation pixels. Therefore, we calculate the DC value of the different backbone networks. The result is shown in Table 1.

**Table 1:** Average Dice’s coefficient (DC) value and prediction speed per picture

Model name	Average DC value	Prediction speed (per/second)
R101FA (our method)	84.51^*^	0.374^+^
IV2RA	83.02	0.538
R101A	82.04	0.519

*The highest average DC value in different models

^+^The fastest prediction speed in different models

*R101FA* residual network-101 with atrous convolution in feature pyramid network, *IV2RA* inceptionV2-residual network with atrous convolution, *R101A* residual network-101 with atrous convolution

From the DC values, we discover that our backbone network R101FA has the highest accuracy. In other words, our model has a better result in burn image segmentation.

To more fully evaluate our model, we chose the different burn images. We chose a total of 120 pictures of different burn depths. There were 20 superficial burns, 50 superficial thickness burns, 40 deep partial thickness burns, and 10 full-thickness burn. Because of a lack of burn images, for the full-thickness burn, we were only able to analyze 10. Then, we calculated the DC values of the different backbone networks.

As shown in Table 2, our model showed better segmentation in the superficial, superficial thickness, and deep partial thickness. Perhaps due to the lack of full-thickness burn images, the results for full-thickness burn in our model were slightly worse than the other models.

**Table 2:** Dice’s coefficient (DC) values of different burn depth in different models

Burn depths	Model name		
	R101FA (our method)	IV2RA	R101A
Superficial	89.7^*^	83.61	77.37
Superficial thickness	85.21^*^	82.52	84.91
Deep partial thickness	84.54^*^	84.44	81.96
Full-thickness burn	81.12	74.56	83.5^*^

*The highest average DC value of this burn depth in different models

*R101FA* residual network-101 with atrous convolution in feature pyramid network, *IV2RA* inceptionV2-residual network with atrous convolution, *R101A* residual network-101 with atrous convolution

On the other hand, there were many patients with extensive burns in the clinical. Therefore, our method needed to demonstrate excellent results in this aspect. Therefore, we selected different burn area size images to evaluate different models. The result is shown in Table 3.

**Table 3:** Dice’s coefficient (DC) values of different burn sizes in different models

Burn sizes	Model name		
	R101FA (our method)	IV2RA	R101A
%TBSA *<* 5%	83.6	86.48	86.63^*^
5% *<* %TBSA*<* 20%	86.13	88.06^*^	84.85
%TBSA*>* 20%	81.27^*^	72.33	74.13

*The highest average DC value of this burn size in different models

*R101FA* residual network-101 with atrous convolution in feature pyramid network, *IV2RA* inceptionV2-residual network with atrous convolution, *R101A* residual network-101 with atrous convolution

As shown in Table 3, the IV2RA has the highest average DC value for 5%< %TBSA < 20% wounds and the R101A has the highest average DC value for the %TBSA < 5% wounds. At the same time, our method has the highest average DC value for the %TBSA > 20% wounds and also has good results in two other sizes.

In order to ensure efficient hospital treatment, the prediction time for each picture must be short. We compared the prediction time of the different backbone networks. As shown in Table 1, our models needed only 0.37 s to predict an image which was the fastest prediction speed.

## Discussion

Burn image segmentation is the first step in the intelligent diagnosis of burn wounds. The precise segmentation is important to the follow-up treatment. In this article, we propose a state-of-the-art segmentation framework to segment the burn images. Compared with the traditional method, this method greatly improves the accuracy of segmentation and contributes immensely to the burn clinic. However, there are still some problems to be solved in this framework. As is known to all, deep learning technology requires a large number of data to ensure the accuracy of the model. In this framework, due to the complexity of data collection and annotation, we only provided almost 1000 pictures to train this model. This makes the model show bad segmentation results in some burn images. In addition, our framework cannot classify the depth of burn wound. In general, the evaluation of wound depth information needs to be combined with the professional knowledge of the doctor, which makes the process of data annotation extremely complicated and difficult for non-professionals to complete. Later, we will collect enough data sets to train the framework to improve the accuracy of the segmentation. Moreover, we will mark the burn wound depth information with the help of the professional, so as to classify the burn wound depth in this framework. And then we will apply our framework to calculate burn area.

## Conclusions

This article proposed a new segmentation framework to segment the burn images based on deep learning technology. In the comparison experiment, we compared the feature extraction capability of different backbone networks. We found that the R101FA backbone network has the best result in accuracy and prediction speed. Finally, we achieved an average of 84.51% accuracy on 150 images. In practice, our method is more convenient than traditional methods and requires only a suitable RGB wound picture. It brings great benefits to the clinical treatment of the hospital.

## Abbreviations

COCO: Common objects in context
DC: Dice’s coefficient
FCN: Fully convolutional network
FPN: Feature pyramid network
IOU: Intersection over union
IV2RA: InceptionV2-Residual Network with Atrous Convolution
R101A: Residual Network-101 with Atrous Convolution
R101FA: Residual Network-101 with Atrous Convolution in Feature Pyramid Network
R-CNN: Regions with Convolutional Neural Network Features
ResNet: Residual network
RoI: Regions of interest
RPN: Region proposal network
TBSA: Total body surface area
